# EXTRACT: interactive extraction of environment metadata and term suggestion for metagenomic sample annotation

**DOI:** 10.1093/database/baw005

**Published:** 2016-02-19

**Authors:** Evangelos Pafilis, Pier Luigi Buttigieg, Barbra Ferrell, Emiliano Pereira, Julia Schnetzer, Christos Arvanitidis, Lars Juhl Jensen

**Affiliations:** ^1^Institute of Marine Biology, Biotechnology and Aquaculture, Hellenic Centre for Marine Research, P.O. Box 2214, 71003 Heraklion, Crete, Greece,; ^2^Alfred Wegener Institute, Helmholtz Centre for Polar and Marine Research, Am Handelshafen 12, D-27570 Bremerhaven, Germany,; ^3^Delaware Biotechnology Institute, Newark, DE 19711, Delaware, USA,; ^4^Max Planck Institute for Marine Microbiology, Celsiusstr. 1, 28359, Bremen, Germany,; ^5^Jacobs University gGmbH, School of Engineering and Sciences, Campus Ring 1, 28759, Bremen, Germany, and; ^6^Disease Systems Biology Program, Novo Nordisk Foundation Center for Protein Research, Faculty of Health and Medical Sciences, University of Copenhagen, Blegdamsvej 3B, DK-2200, Copenhagen, Denmark

## Abstract

The microbial and molecular ecology research communities have made substantial progress on developing standards for annotating samples with environment metadata. However, sample manual annotation is a highly labor intensive process and requires familiarity with the terminologies used. We have therefore developed an interactive annotation tool, EXTRACT, which helps curators identify and extract standard-compliant terms for annotation of metagenomic records and other samples. Behind its web-based user interface, the system combines published methods for named entity recognition of environment, organism, tissue and disease terms. The evaluators in the BioCreative V Interactive Annotation Task found the system to be intuitive, useful, well documented and sufficiently accurate to be helpful in spotting relevant text passages and extracting organism and environment terms. Comparison of fully manual and text-mining-assisted curation revealed that EXTRACT speeds up annotation by 15–25% and helps curators to detect terms that would otherwise have been missed.

**Database URL**: https://extract.hcmr.gr/

## Introduction

The annotation of microbial samples with rich metadata is an essential prerequisite to comparative data analysis, and it is crucial that its content, syntax and terminology are standardized. In addition to enabling efficient search, discovery, and retrieval operations, high-quality metadata can, itself, be the subject of meaningful analyses. This is particularly true in metagenomics, where microbial community composition and function are strongly tied to the community’s environment type and state at the time of sampling.

To address this need, the microbial and molecular ecology research communities have initiated the development of standards, checklists and detailed guidelines for reporting sample metadata (1). These include metadata such as geographic location, date and time, sampling procedure and sampling environment. The value of quantitative parameters in numerical approaches is clear; however, qualitative descriptors too, have much to offer. Qualitative descriptions of, e.g. the environment from which a sample was obtained are of particular interest, as they can provide researchers with additional details that often cannot easily be captured in quantitative form. However, even when descriptions are present, they are of limited use if they exist only as unstructured text. Annotation of samples metadata with ontology terms complements free-text descriptions with semantically controlled descriptors; these descriptors avoid the confusion caused by synonyms and have explicitly defined relations to other terms.

The Environment Ontology (ENVO; http://environmentontology.org)—a project linked to the Genomics Standards Consortium (GSC; http://gensc.org)—specifically deals with the challenge of representing non-quantitative but structured knowledge about environment types (2). It provides researchers with a semantically coherent, concise and structured environment description resource to explicitly capture the source environments of, e.g. microbiomes and natural history museum specimens. ENVO terms fall into three distinct hierarchies, namely biome, environmental feature and environmental material. To fully describe the environment of a sample, GSC recommends that the environment annotation of a sample should feature at least one term from each hierarchies of the ontology. Also, microbiomes associated with certain parts of specific organisms can be described by combining ENVO terms with taxa from NCBI Taxonomy (3) and terms from ontologies that model anatomy (4, 5) and disease phenotypes (6, 7). However, making such annotations requires curators to be familiar with the terminologies used, and manually assigning terms to the existing backlog of samples will be a major effort.

Named Entity Recognition (NER), i.e. automatic identification of terms mentioned in text, can assist the standards-compliant reporting of sampling environments in two primary ways. Although most work on biomedical NER has focused on recognition of gene and protein names (8), methods also exist for recognizing terms more relevant to sample annotation, namely organisms (9, 10), tissues (11, 12), diseases/phenotypes (13–17) and environments (18). NER can be used to suggest terms based on existing free-text fields in sequence/sample repositories or the literature associated with the samples.

The Reflect (19) tool demonstrates how NER can be employed via a user-friendly interface to provide readers with easy access to background information about the entities mentioned in a web document. It does so by presenting a popup with additional information about one entity of interest at a time. This approach, however, is not ideally suited for curators, who need the ability list multiple identified terms, map them to identifiers, and extract them in a structured form.

Here we present the EXTRACT system, which combines components of the previously published ENVIRONMENTS (18), SPECIES/ORGANISMS (10), TISSUES (12), DISEASES (17) and Reflect (19) systems to identify and extract standard-compliant terms for annotation of metagenomic records and other samples. The system was evaluated in the BioCreative V Interactive Annotation Task (IAT).

## Materials and methods

EXTRACT is an interactive tool that is designed to help curators more efficiently identify environment descriptors, organisms, tissues and diseases mentioned in text and annotate these using ontology/taxonomy terms. The system consists of three separate components: 1. a server that performs the actual NER task, 2. a bookmarklet that allows users to easily submit text from a web page to the server and 3. a popup that allows users to inspect the identified terms and extract these annotations in tabular form.

### Named entity recognition

The NER component of EXTRACT is based on a combination of several previously published systems. The core is a highly optimized dictionary-based tagger engine, implemented in C ++, which is able to do flexible matching of a dictionary with millions of names against thousands of abstracts per second (10). The fast performance of the tagger engine makes it well suited for real-time applications, such as the interactive annotation task.

Using a variety of different dictionaries, we have previously used this tagger to identify names of species and other taxa (10), tissues and other anatomical entities (12), diseases (17) and last but not least environments (18). The quality of the tagging results for species and environments has previously been evaluated on gold-standard corpora consisting of Medline abstracts and of Encyclopedia of Life (20) species summary pages, respectively (10, 18). Counted at the level of individual mentions, the SPECIES/ENVIRONMENTS taggers showed precisions of 83.9% and 87.8%, recalls of 72.6% and 77.0%, and F_1_ scores of 78.8% and 82.0%. The quality of the NER of tissues and diseases has not been benchmarked directly; however, these NER components have shown to give good results when used for co-mention-based extraction of protein–tissue and protein–disease associations (12, 17).

As all the dictionaries needed for NER on this project have been optimized for use with the same tagger engine, the EXTRACT server backend consists of a single tagger instance that uses the combined dictionary of the four previously published NER systems. To expose the tagger as a RESTful web service, we used the Python web resource framework developed as part of the Reflect system (19). We expanded the framework with new API calls for presenting tagging results in the form of a popup. The documentation for the API can be found on the EXTRACT help page (https://extract.hcmr.gr/#extract_help).

### Bookmarklet

The primary way for users to interact with the EXTRACT server is via a so-called *bookmarklet*, which is a browser bookmark containing a small JavaScript. The EXTRACT bookmarklet is available at https://extract.hcmr.gr/and is installed by simply dragging and dropping it into the bookmark bar of the web browser. The bookmarklet, much like a browser extension, can be used on most HTML pages, including PubMed abstracts, full-text journal articles and web pages from various sequence/sample databases. There are two ways to use it.

**Figure 1.**
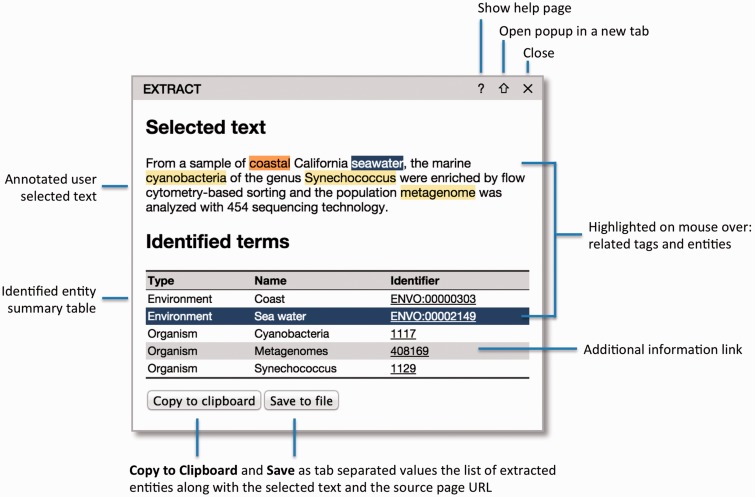
The EXTRACT popup. Hovering the mouse cursor over the text tags or the table rows enables users to visually inspect which words have been identified as which entities. To allow for easy collection of annotations in tabular form, e.g. in an Excel spreadsheet, two buttons allow the user to either copy the information to the clipboard or save it to a tab-delimited file. When doing so, the selected text and the address of the source webpage are also included for provenance.

First, to extract annotations, the user can select some text of interest within a web page and click the bookmarklet. The selected text will then be sent to the NER server, and a popup will appear with the identified terms (Figure 1). By processing only the user-selected text clause, EXTRACT allows for fine-grained, sample-specific term extraction from, for example, articles describing multiple samples from different sources. The functionality of the popup is explained in detail in the next section.

**Figure 2.**
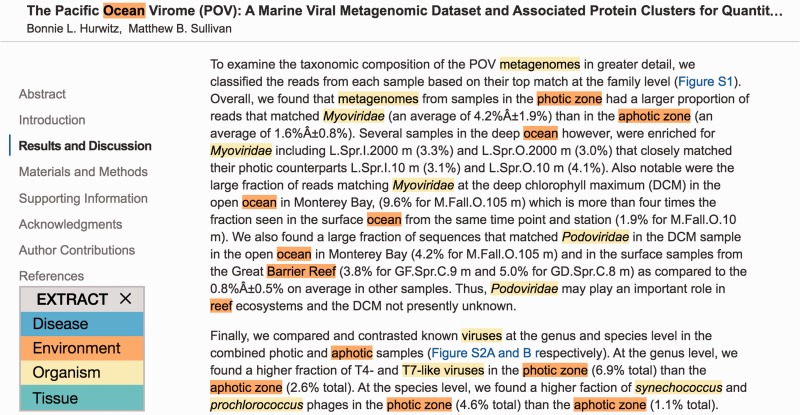
EXTRACT highlighting of terms. To quickly identify metadata-relevant pieces of text in a larger document, users may perform a full page tagging. The highlighted entities can indicate candidate segments for subsequent inspection and term extraction. As shown, identified organisms mentions are highlighted in yellow and environment descriptors in orange. The example shows an excerpt of reference (21).

Second, the user can click the bookmarklet without selecting any text. In this case, the entire HTML page will be sent to the NER server for processing, and the recognized names will be highlighted within the page, using different colors for different types of entities (Figure 2). This functionality can help curators to more quickly identify the relevant parts of a page, from which annotations can subsequently be extracted by selecting them and again clicking the bookmarklet.

### Popup

The final component of the EXTRACT system is the popup (Figure 1), which enables users to inspect the terms identified within a text selection and collect the corresponding annotations in tabular form.

The popup consists of two main parts: (1) the selected text with the identified names marked and (2) a table listing the corresponding ontology/taxonomy terms. When hovering the mouse cursor over a highlighted name in the text, the corresponding table rows are highlighted and vice versa. This allows users to visually inspect the textual support for each term.

To enable users to easily collect annotations in tabular form, e.g. in an Excel spreadsheet, two buttons allow the table to either be copied to the clipboard or saved to a tab-delimited file. When doing so, the selected text and the address of the source web page are included as additional columns for provenance.

The title bar of the popup allows it to be moved and contains three icons in the top-right corner: help (?), pop-out (⇑) and close (✗). Clicking the pop-out icon opens a full-page version of the popup in a separate browser tab. The functionality of the full-page version is identical to that of the popup, but provides a better overview than the popup when a large text selection has been processed or many entities have been identified. Whereas only one popup can be visible at a time in the compact mode, many popups can be open in the full-page view as separate browser tabs. This facilitates a two-stage workflow appropriate for large documents: users can first go through a document, identify relevant sections and open the popups as separate tabs and subsequently go through all the tabs to extract the annotations.

Because the popup makes use of browser features introduced in HTML5, only recent browsers are supported. EXTRACT has been tested and found fully functional in the latest versions of Google Chrome, Mozilla Firefox, Safari and Opera. EXTRACT is also usable in the latest version of Internet Explorer, although the possibility to save information as a tab-delimited file is not available.

## Results and discussion

The EXTRACT system was evaluated in the BioCreative V Interactive Annotation Task (IAT). Unlike most BioCreative tasks, the aim of IAT is not purely to evaluate the precision and recall of various text-mining methods, but rather to assess the utility and usability of text-mining-assisted curation systems (22). Unlike many systems that have previously participated in the IAT (23–26), EXTRACT is not designed for annotation of text corpora. Instead, the goal is to help curators make structured environment annotations of metagenomic records and other samples.

### Evaluation of ease of use and interface functionality

To evaluate the user experience for new users with limited, if any, prior training in using EXTRACT, feedback was collected from ten anonymized *partial evaluators*, who have experience in data curation but different scientific backgrounds. They performed a sequence of predefined tasks and commented on their experience by filling in a survey assessing EXTRACT both generally as a software system and specifically as a curation tool. The survey covered many aspects of using EXTRACT, including the installation procedure, the clarity and intuitiveness of the user interface, the ease and usefulness of individual features, and the applicability in helping curators identify and extract annotations. Adhering to the IAT principle of prioritizing assessment of the user experience, emphasis was given in recording the ease and the confidence in performing each task as well as the helpfulness of the documentation and error messages.

All but one evaluator found it easy or very easy to install EXTRACT. They all successfully used it to tag a web page, understood that different colors correspond to different types of terms, and found this feature useful. Four of the ten evaluators encountered false positives or false negatives, two of whom noted that the extracted terms were generally correct. They all found it easy or very easy to run EXTRACT on a selection to get the popup, and all but one also found it easy to save the results from the popup. The content of the download files surprised many evaluators, because it contains additional columns not shown in the popup. This was, however, a positive surprise, with most evaluators noting that they found the additional information useful. Not all evaluators found it easy to understand the connection between synonyms and terms, though, especially not in the download files.

The evaluators all found the documentation to be helpful or very helpful. When asked to find the answer to a specific question, they rated this task as neutral to very easy. Only four evaluators encountered error messages, two of whom found them helpful. All but one evaluator, who was neutral on the topic, found EXTRACT to be straightforward to use, at least after looking at the documentation.

At the end of the survey, all partial evaluators rated EXTRACT as a positive or very positive experience and found it to be helpful for extracting species and environments from text in the form of controlled vocabulary terms. On the question as to whether they would recommend EXTRACT to a colleague, the evaluators gave an average score of 8.3 on a scale from 0 (Not at all likely) to 10 (Extremely likely).

### Utility for metadata annotation of metagenomic samples

Two of the partial evaluators served also as full evaluators, whose task was to assess the utility of the EXTRACT system for actual curation. Their assessment was conducted in the context of annotating sequences from the MetagenomesOnline database (27) and from marine metagenomic records in other public databases based on their associated full-text articles. Specifically, they were asked to test if EXTRACT can help them locate environment information about a sample, and if it can accelerate standards-compliant metadata annotation of metagenomic samples with environmental feature, material and biome terms. To this end they were provided with simple annotation examples and curation guidance in the documentation. The full evaluators were not given a predefined set of samples and pertinent documents to annotate purely for the purpose of the BioCreative IAT. Instead, they were asked to test EXTRACT within their usual workflow on records that they would in any case annotate as part of their normal curation tasks. The simple EXTRACT tabular annotation format facilitated this as it can easily fit into most existing workflows.

The speedup of the annotation process is difficult to assess for several reasons. First, the time needed for curation varied greatly between papers and depended as much on how the information was organized within the paper as it did on the number of terms that needed to be annotated. Second, some of the most time-demanding aspects of the curation process fall outside the scope of EXTRACT, such as the annotation of geographic coordinates and other numeric metadata. To estimate the speedup attained by using EXTRACT, we focused on the times reported purely for term annotation of samples by the two full evaluators, who extracted environmental terms from two separate sets of eight full-text articles. This suggests a speedup in the range of 15–25%. The main timesaver was that EXTRACT saved the curators the hassle of having to look up the ENVO identifier for every term separately, which was indeed what the design of the popup aimed to address.

Since no NER method has 100% recall, EXTRACT is bound to miss some terms found by the curators, and the full-page tagging functionality could not eliminate the need to read through the entire paper. This is a major reason why the speed-up is not greater than what we report above. However, on the positive side, both full evaluators noted that EXTRACT helped them find terms that they had not thought of themselves and which would thus otherwise have been missed.

Whereas EXTRACT cannot, and was never intended to, replace curators, the results of the full evaluation shows that the tool can help the curators make more complete and accurate annotations, while providing a modest speed-up.

### Improvements to EXTRACT

Besides assessing the functionality and usefulness of EXTRACT, the evaluators also provided the development team with useful suggestions for improvements, most of which have already been implemented.

As requested by many evaluators, we have now added a color legend for the types of terms when tagging an entire page. This is only one of two improvements to the full-page tagging; the other is that one can now click on or hover over any of the recognized terms to see the corresponding popup. This second feature was added in response to some evaluators finding it cumbersome to have to select text and click the bookmarklet again after having tagged a page.

The input from the evaluators also prompted us to improve the consistency of the color schemes used in the full-page tagging and in the popup. In the new version of EXTRACT, the terms in the selected text are highlighted using the same term-type-specific color scheme used when tagging an entire page. We decided against using the same color scheme in the table too, as this results in reduced readability and makes the highlighting of the mouse-over functionality less clear. The new version of the popup is shown in Figure 1.

The tab-delimited information, which can be either copied to the clipboard or saved as a file, has been extended with an additional column that contains the matched substrings within the text. When saving this information as a file, we now also provide a header row; this is not included when copying the information into the clipboard, since it would lead to inconvenient, redundant header rows when gathering annotations in a spreadsheet.

Besides the inclusion of a color legend, the most commonly requested improvement from the evaluators was to separate ENVO terms into three types: biome, feature and material. Whereas we fully understand why this requested functionality would be useful, currently, we unfortunately cannot implement it. Unlike Gene Ontology (28), which separates molecular function, biological process and cellular component terms into separate namespaces, ENVO uses a single namespace for all terms. We consider solving this by inferring the type of each term from the logic relationships in the ontology; however, this is not currently possible for all terms and will thus require changes to ENVO itself.

Finally, in light of the positive feedback from the evaluators, we have begun work on expanding the scope of EXTRACT beyond annotation of environment metadata. Given the generality of the interface and workflow, it should be applicable to many other tasks, including extraction of drug–target associations and annotation of protein function and localization. To support such use cases, we are extending the dictionary to include also names of genes, proteins, small-molecule compounds, and Gene Ontology terms. Although still under development, this new functionality can already be previewed via the EXTRACT2 beta bookmarklet, which is available from the EXTRACT website.

## Conclusions

The tool was generally well received by the evaluators, although there are still improvements to be made. EXTRACT was designed to be a generic tool that provides annotations in a simple tab-delimited format, which should make it easy to utilize in existing spreadsheet-based curation workflows. A generic tool obviously cannot bring the same benefits as a tool customized to integrate optimally into the workflow of a specific database. To allow developers to tightly integrate EXTRACT with other software, the named entity recognition server is exposed as a web service that accepts both HTML and plain text. This also enables the use of EXTRACT as the NER component of a larger NLP system.
